# Organophosphate Insecticides Resistance in Field Populations of House Flies, *Musca domestica* L.: Levels of Resistance and Acetylcholinesterase Activity

**DOI:** 10.3390/insects13020192

**Published:** 2022-02-11

**Authors:** Yasser Abobakr, Faisal I. Al-Hussein, Alaa E. Bayoumi, Ali A. Alzabib, Ali S. Al-Sarar

**Affiliations:** 1 Department of Plant Protection, College of Food and Agriculture Sciences, King Saud University, Riyadh 11451, Saudi Arabia; yawadallah1@ksu.edu.sa (Y.A.); soon-f@hotmail.com (F.I.A.-H.); ahs222950@gmail.com (A.A.A.); 2 Department of Animal Pests, Sabahia Research Station, Plant Protection Research Institute, Agricultural Research Center, Alexandria 21616, Egypt; 3 Department of Plant Protection, Faculty of Agriculture, Ain Shams University, Shoubra El-Kheima, Cairo 11241, Egypt; alaaeb@agr.asu.edu.eg

**Keywords:** *Musca domestica*, diazinon, fenitrothion, insecticide resistance, acetylcholinesterase

## Abstract

**Simple Summary:**

The house fly, *Musca domestica* L., is an important medical and veterinary pest associated with humans and livestock. Management of house flies has relied extensively on chemical control. The inappropriate use of insecticides has led to resistance worldwide. Insecticide resistance is one of the critical challenges in applied pest management. Resistance is defined as an inherited potential of a population to tolerate an insecticide dosage that is lethal for the majority of individuals of a susceptible population of the same species. The development of resistance is producing significant environmental threats, such as adverse effects on non-target organisms and environmental poisoning. Therefore, monitoring the resistance status of *M. domestica* field populations is considered critical for avoiding these environmental threats. In the present study, we found high levels of resistance in the house fly field-collected populations from Riyadh, Saudi Arabia to organophosphate insecticides, diazinon, and fenitrothion. Therefore, the use of organophosphate (OP) insecticides should be stopped and replaced with novel insecticides having different modes of action in the house flies control programs.

**Abstract:**

The house fly, *Musca domestica* L., is an important medical and veterinary pest associated with humans and livestock. Management of house flies has relied extensively on chemical control. In this study, we report on the resistance of house fly field-collected populations to diazinon and fenitrothion OP insecticides in Riyadh, Saudi Arabia. The diazinon and fenitrothion median lethal dose (LD_50_) values against adult female *M. domestica* field-collected populations were significantly higher than those of the laboratory (LAB) strain. Different levels of resistance were detected in all field-collected populations toward the two OP insecticides. The resistance ratios for diazinon ranged from 62.47 to 309.78, while there were 53.08 to 261.24 for fenitrothion in the eight field-collected populations. The specific activity of acetylcholinesterase (AChE) in all field populations was significantly (*p* < 0.05) higher than that in the LAB strain. In vitro diazinon and fenitrothion median inhibitory concentration (IC_50_) values of LAB strain AChE activity were significantly (*p* < 0.05) lower than those for field-collected populations. This study found high levels of resistance in the house fly field-collected populations to diazinon and fenitrothion. Replacing these two insecticides and any other OPs with novel ones that have different modes of action is an urgent need in the insect-vector control programs in Riyadh, Saudi Arabia. An altered AChE enzyme of *M. domestica* field populations might be partially responsible for the developed resistance. Monitoring of insecticide resistance development in *M. domestica* populations and a better understanding of its mechanisms are needed to design operative management strategies for controlling the house flies.

## 1. Introduction

The house fly, *Musca domestica* L., is likely the most commonly distributed medical and veterinary insect pest associated with humans and its domesticated animals. House flies reproduce in all types of organic matter, and the adult stage is more dangerous than the larval stage because of its high ability to move and concurrent contact with animals, human beings, and decaying or rotting materials. They have been identified as mechanical vectors transmitting more than 100 pathogenic and parasitic species (bacteria, protozoans, virus, and parasitic nematodes) that cause a great number of diseases (e.g., amoebic dysentery, salmonellosis, bacillary dysentery, Coxsackie, cholera, polio, hepatitis, typhoid, and paratyphoid) to humans, poultry, and livestock [1,2,3,4,5]. 

Management of house flies has relied extensively on chemical control. The inappropriate and repetitive use of insecticides has led to resistance worldwide [6,7,8]. Insecticide resistance is one of the critical challenges in applied pest management. Resistance is defined as an inherited potential of a population to tolerate an insecticide dosage that is lethal for the majority of individuals of a susceptible population of the same species. The development of resistance is producing significant environmental threats, such as adverse effects on non-target organisms and environmental poisoning. It is estimated that more than 500 species of Arthropoda developed resistance to one or more pesticides [9,10,11]. Therefore, monitoring the resistance status of *M. domestica* field populations is considered critical for the development of realistic and effective control programs [12]. Unfortunately, there is no periodic monitoring of insecticide resistance in house fly populations in Riyadh, Saudi Arabia. However, different levels of resistance to the OP insecticides, diazinon (6.8–72 folds) and fenitrothion (7–27 folds), were recorded in house fly field populations in Riyadh [13,14]. 

OP insecticides are esters of phosphoric acid (or its sulfur analogs) used to control many insect pests worldwide in great amounts [15]. They were primarily introduced as pest control agents over 60 years ago [16]. The initial success of OPs was principally based on their high toxicity, rapid environmental degradation, and high biological specificity [17]. OP insecticides cause toxicity through their high affinity for binding to the active site and inhibiting the enzyme acetylcholinesterase (AChE, EC 3.1.1.7), a neural enzyme responsible for the degradation of the excitatory neurotransmitter acetylcholine (ACh) to choline and acetate, thus terminating nerve impulse transmission at cholinergic synaptic clefts. Inhibition of AChE extends the residence time of ACh at synapses, resulting in hyperexcitation and death [18,19,20,21]. The biochemical specificity of OPs has resulted in the resistance development in numerous insect pests [22,23]. 

In the house fly control program in Riyadh, OP insecticides are still in use [24]. This study was conducted with field-collected populations of house flies to assess their resistance status to OP insecticides, diazinon and fenitrothion, in Riyadh slaughterhouses and vegetable markets. In addition, we evaluated the specific activity of AChE in both field-collected populations and LAB strain as well as the inhibitory effect of both insecticides against AChE. 

## 2. Materials and Methods

### 2.1. Insects

The OPs susceptible *M. domestica* laboratory strain (LAB) was provided by the Public Health Pests Laboratory (PHLP-Jeddah, Jeddah, Saudi Arabia.) This strain was collected from sites that have not been exposed to insecticides and have been bred in the laboratory since 2006. Field populations were collected from eight sites: Sa’ada (SAS), Mowanneseiah (MOS), Mansoureiah (MAS), North (NOS), and West (WES) slaughterhouses and Azizeiah (AZM), Badea’ah (BAM), and Rabwah (RAM) wholesale vegetable markets in Riyadh city, Saudi Arabia (Table 1). From each collection site, adult house flies were collected by a sweep net. Each population was placed in a cage (50 × 50 × 50 cm^3^) in the laboratory and kept at 25 ± 2 °C and in a 12:12 light/dark photoperiod. The diet for adult flies consisted of sugar and powdered milk at a ratio of 1:1 and it was replaced every two days. A wet cotton wick was used as a water source, and it was moistened daily. An artificial diet of wheat bran, yeast, milk, and water in a proportion of 20:1:2:20, respectively, was prepared for the oviposition and larvae diet source.

### 2.2. Insecticides

**Figure 1:**
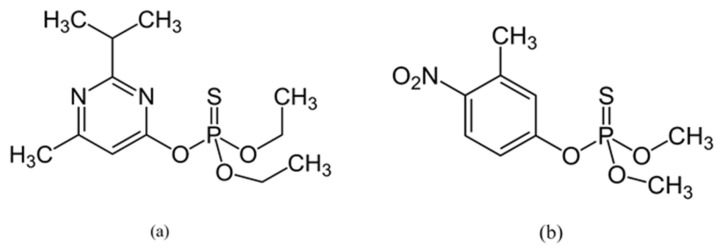
Chemical structure of: (**a**) diazinon; (**b**) fenitrothion.

Two organophosphate insecticides were used in this study: (i) diazinon (*O*,*O*-Diethyl *O*-[4-methyl-6-(propan-2-yl) pyrimidin-2-yl] phosphorothioate, 98.5%, Figure 1a) and (ii) fenitrothion (*O*,*O*-dimethyl *O*-(3-methyl-4-nitrophenyl) phosphorothioate, 98.5%, Figure 1b from Sigma-Aldrich Chemie GmbH, Taufkirchen, Germany. 

### 2.3. Bioassays

Adult female house flies (3–5-day-old) were anesthetized with CO_2_, and 1.0 μL of the insecticide diluted in acetone was applied on the thoracic notum of each fly using a 25 µL Hamilton syringe via a Hamilton micro applicator (Hamilton Company, Bonaduz, Switzerland). For each insecticide, five different dilutions were tested to obtain mortality percentages between 5% and 95%. Similar doses of diazinon and fenitrothion were applied to the following populations: LAB (1, 2, 4, 6, and 8 ng/♀), RAM (50, 100, 200, 400, and 600 ng/♀), AZM and BAM (100, 300, 500, 700, and 900 ng/♀), MOS and SAS (700, 900, 1100, 1300, and 1500 ng/♀). The following populations received different doses of the two insecticides: MAS (diazinon: 500, 700, 900, 1100, and 1300 ng/♀; fenitrothion: 700, 900, 1100, 1300 and 1500 ng/♀), NOS (diazinon: 100, 200, 400, 600, and 800 ng/♀; fenitrothion: 700, 900, 1100, 1300, and 1500 ng/♀), and WES (diazinon: 100, 300, 500, 700, and 900 ng/♀; fenitrothion: 900, 1100, 1300, 1500, and 1700 ng/♀). Groups of 20 flies per concentration were replicated 3 times (*N* = 360). Control groups received acetone alone. After topical application, flies were kept in 250 mL plastic containers covered with tulle cloth and secured with rubber bands. A 10% sugar-soaked cotton was placed at the bottom of each container. Mortality was recorded 24 h after treatment, and flies were considered dead if they were on their backs without any movement when disturbed. 

### 2.4. AChE Activity Assays

The enzyme activity was determined using Ellman’s method [25] with slight modifications using 3–5-day-old adult female house flies. Briefly, heads of five flies from each population were pooled and homogenized in 1 mL of ice-cold sodium phosphate buffer (0.1 M Na_2_HPO_4_, pH 7.8) with a tissue homogenizer (Cole-Parmer, Vernon Hills, IL, USA). The homogenates, after filtration, were used as the enzyme source. In the reaction tube, 1% Triton X-100 (1450 µL), 5,5′-dithiobis-(2-nitrobenzoic acid) (100 µL), enzyme homogenate (250 µL), and acetylthiocholine iodide (250 µL) were added. The enzyme sensitivity to inhibition was assessed by measuring its activity in the presence of different concentrations of diazinon or fenitrothion. Six diazinon or fenitrothion concentrations were used in triplicate to get the IC_50_ (concentration needed to inhibit 50% of enzyme activity) values. These concentrations were 0.25, 0.5, 0.75, 1, 1.25, and 1.5 µg/mL for the LAB population while they were 25, 50, 100, 200, 300, and 400 µg/mL for field populations. Changes in absorbance were recorded by a Jenway 6705 UV/Visible scanning spectrophotometer (Bibby Scientific Ltd., Essex, UK) at 405 nm.

### 2.5. Statistical Analysis

Dose-mortality and dose-AChE inhibition data were used to calculate the lethal and enzyme inhibitory dose values, respectively. The correction of mortality data was done according to Abbott’s formula [26]. The data were subjected to probit analysis [27] using LdP software (Ehabsoft, Cairo, Egypt). The LD_50_, IC_50_, 95% confidence limits, slope, resistance ratio (RR), and inhibition resistance ratio (IR) were calculated. The chi-squared (χ^2^) test was used to evaluate the goodness-of-fit of the model to the data. Variances among LD_50_ or IC_50_ values were considered significant if their confidence limits (95%) did not overlap [28]. Pearson correlation coefficient (*r*) was calculated as a measure of linear correlation between diazinon and fenitrothion LD_50_ values_,_ AChE activity, and LD_50_ values of the two OPs insecticides, and between IC_50_ and LD_50_ values or AChE activity.

## 3. Results

### 3.1. Susceptibility of House Flies to Diazinon and Fenitrothion

The LD_50_ values of adult female *M. domestica* field populations were significantly higher than that of the LAB strain for diazinon (Table 2) and fenitrothion (Table 3). Diazinon LD_50_ of the MOS population was significantly higher than those of the LAB strain and the field-collected populations, except for SAS. Likewise, there is no significant difference in the LD_50_ values between SAS and MAS populations. Also, diazinon LD_50_ values of WES, AZM, BAM, and NOS populations were insignificantly different. For fenitrothion, LD_50_ of WES was significantly higher than those of the LAB strain and the rest of the populations except SAS. The lowest LD_50_ was recorded for RAM, which was significantly the lowest among those of the other field populations for both diazinon and fenitrothion. Different levels of resistance were detected in all field populations toward the two OP insecticides. The most diazinon-resistant population was MOS (RR = 309.78-fold), while WES was the most fenitrothion-resistant population (RR = 261.24-fold). RAM was the least resistant population to diazinon and fenitrothion, with RRs of 62.47- and 53.08-fold, respectively. The LD_50_ values of diazinon and fenitrothion were found to be strongly positively correlated (*r* = 0.7, *p* = 0.037).

### 3.2. Activity of AChE and Inhibitory Effect of Diazinon and Fenitrothion

The specific activity of AChE was assessed in both LAB strain and field populations, and the results are displayed in Table 4. The enzyme activity in all field populations was significantly higher than that in the LAB strain, suggesting the altered field populations' AChE enzyme properties compared with that of the LAB strain. While a strong positive correlation between AChE activity and fenitrothion LD_50_ values were observed (*r* = 0.7, *p* = 0.035), a moderate positive correlation between AChE activity and diazinon LD_50_ values was found (*r* = 0.51, *p* = 0.16) although the latter correlation is not significant. 

In vitro assays were performed to determine the inhibitory effect of diazinon and fenitrothion to LAB strain and field-collected house fly AChE. The IC_50_ values of diazinon (Table 5) and fenitrothion (Table 6) for the LAB strain were found to be 0.77 and 0.99 µg/mL, respectively; and these values were significantly lower than those of the field population. MOS population displayed the greatest IC_50_ value (172.59 µg/mL) for diazinon, which was 224.14 times compared to that of the LAB strain (Table 5). The lowest diazinon IC_50_ among field populations was recorded for WES (48.34 µg/mL) equal to 62.77 times that of the LAB strain. For fenitrothion, the highest and the lowest IC_50_ values in field populations were recorded for MAS (307.76 µg/mL) and RAM (71.66 µg/mL) populations, which were 310.86 and 72.38 times compared with those of the LAB strain, respectively (Table 6). The LD_50_ and IC_50_ values of diazinon were found to be strongly positively correlated (*r* = 0.88, *p* = 0.002). Similar strong positive correlation was noticed between LD_50_ and IC_50_ values of fenitrothion (*r* = 0.8, *p* = 0.018).

## 4. Discussion

This study was conducted to evaluate the resistance of field-collected populations of the house fly *M. domestica* in Riyadh city, Saudi Arabia toward two OP insecticides, diazinon, and fenitrothion. The insect populations should be considered resistant if they develop tenfold resistance to insecticides [29]. Accordingly, the eight field populations of *M. domestica* collected from slaughterhouses and vegetable markets displayed different levels of resistance to diazinon (RR = 62.47–309.78 folds) and fenitrothion (RR = 53.08–261.24 folds). These differences in the resistance levels among the field-collected populations could be attributed to the different patterns of exposure (insecticide type, application method, frequency, and the period of exposure). However, Hafez [14] reported low to moderate resistance levels to fenitrothion (7–27 folds) in house fly field populations collected from dairy farms around Riyadh. These low or moderate levels of resistance to fenitrothion may be attributed to the lack of extensive exposure to insecticides in the dairy farms, unlike the situation of slaughterhouses and vegetable markets in our study. In 2015, an earlier study reported lower ratios of resistance (6.8–72 folds) to diazinon compared with our results on house fly field populations collected from the same slaughterhouses in Riyadh; and the researchers predicted that the resistance will constantly increase if the same or similar insecticides are used to control the public health insects in Riyadh [13]. Our results indicate 3, 4, 16, and 31 times increases in the resistance of WES, SAS, NOS, and MOS populations, respectively, to diazinon over six years compared to the previous study [13]. The Saudi Food and Drug Authority (SFDA) [24] revealed that the conventional insecticides (OPs, pyrethroids, and neonicotinoids), including fenitrothion, are currently used for controlling public health insects, including house flies. The extensive use of conventional insecticides, such as OPs in slaughterhouses and vegetable markets, has led to an emergence of strong resistance levels among the field populations toward these insecticides, declining their efficacy. Our findings revealed that the NOS population recorded the lowest resistance level to diazinon among slaughterhouse populations, while the SAS population was one of the most resistant populations. Similar results were reported six years ago, where the population collected from NOS was the lowest resistant to diazinon, while the SAS population was the highest [13]. This agreement in the results may indicate that the same control measures were followed in each slaughterhouse during this period between the two detections. The population of MOS was the second-lowest resistant population (9.9 folds) to diazinon in 2015 [13]. In the present study, the resistance level of this population increased by 31 times to become the highest (309.78 folds) among all investigated populations. This remarkable increase in the level of resistance points out the high rate of selection pressure through the intensive use of conventional insecticides, especially OPs, in this site. Previous studies demonstrated that resistance to OP insecticides occurs in *M. domestica* and it is a worldwide challenge [12,30,31,32]. In accordance with our results, high levels of house fly resistance to fenitrothion were recorded in different strains and countries, such as the Şanlıurfa 2004 strain from Turkey (50.37 folds), the Danish strain (100–400 folds), strains from Taiwan (12.47–134.79 folds), the Japanese strain Akita-f (3500 folds), and the Hans strain from Germany (>6700 folds) [33,34,35,36,37].

The positive correlation between toxicity values of different insecticides may indicate the presence of cross-resistance among them [38]. Here, the recorded strong positive correlation between LD_50_ values of diazinon and fenitrothion may suggest the cross-resistance between them. The cross-resistance in house fly was reported for different insecticides [39]. 

Early detection of insecticide-resistant populations may efficiently diminish the environmental, operational, and financial costs associated with house fly management. Unfortunately, no insecticide resistance monitoring program of house fly populations is available in Riyadh city at present. An operative resistance management program would bring new insights into the development of resistance at the studied sites. Therefore, the application of systematic surveys on breeding sites would be informative in order to establish effective control strategies against house fly populations and avoid future control failures. Moreover, insect growth regulators (e.g., pyriproxyfen) that showed efficacy against the house fly field populations in Riyadh [40] should be included in the control programs. In addition, the rotation of insecticides with different modes of action is needed to reduce the rapidity of resistance development in house fly field populations.

AChE enzyme plays a vital role in the nervous system of insects, where it catalyzes the hydrolysis of the neurotransmitter, ACh, at the cholinergic synaptic gaps. OP insecticides inhibit AChE causing the desensitization of ACh receptors and leading to the disruption of neurotransmission. Modified AChEs, which are less sensitive to OP insecticides, are known to confer insecticide resistance [41]. The assessment of AChE specific activity in LAB strain and field populations revealed significant differences between them, suggesting altered biochemical properties of field populations’ AChE enzyme compared with that of LAB strain. In the present study, there were strong and moderate positive correlations between AChE activity and LD_50_ values of fenitrothion and diazinon, respectively. These positive linear correlations could be considered an indicator of the involvement of AChE, at least partially, in the resistance to these insecticides. However, the variation in the strength of correlations may be due to the different patterns of control treatments among the selected sites. All field-collected populations of house fly exhibited reduced sensitivity of AChE to inhibition by diazinon and fenitrothion, compared with that of the LAB strain. A strong linear correlation between IC50 and LD50 values for diazinon and fenitrothion is expected because AChE is the primary target for the two insecticides. Moreover, this positive correlation suggests the involvement of AChE in the developed resistance in the investigated populations of the house fly. It was reported that variations of AChE were related to resistance in house flies [42,43]. AChE reduced sensitivity to inhibition has been found to confer resistance in house flies [44,45,46]. The results of Kim and Boo [47] boast that OP resistance in house flies has resulted from the change in the sensitivity of AChE to OP insecticides. Kozaki et al. [48] found a difference in the AChE residual activity inhibited by fenitroxon between susceptible and resistant house fly strains and they associated the mutations at Gly342 and Tyr407, located in the active site of the enzyme, with the insensitivity to fenitroxon. In addition, the biochemical properties of AChE in a propoxur-resistant house fly were different compared with those of the susceptible strain enzyme [49]. Point mutations were identified in the *ace* gene of the propoxur-resistant fly strain [50]. Consequently, it is suggested that the *ace* gene in the Riyadh field-collected populations might also be mutated, resulting in different levels of resistance to diazinon and fenitrothion insecticides.

## 5. Conclusions

In conclusion, the field-collected populations from different slaughterhouses and vegetable markets in Riyadh, Saudi Arabia, displayed high levels of resistance to the OP insecticides, diazinon, and fenitrothion. Hence, replacing these two insecticides and any other OPs with novel ones that have different modes of action is an urgent need in insect-vector control programs. Monitoring of *M. domestica* populations in Riyadh for insecticide resistance should be carried out for current and newly applied insecticides to evaluate their efficacy. A better understanding of the mechanisms of insecticide resistance development and the evolution in *M. domestica* populations is needed to develop effective management strategies for controlling house fly populations. Therefore, further studies should be performed involving biochemical and molecular mechanisms.

## Figures and Tables

**Table 1:** Collection sites of house fly field populations in Riyadh city.

Site Name	Activity	Coordinates	
		Latitude	Longitude
Sa’ada (SAS)	Slaughterhouse	24.70812 N	46.85459 E
Mowanneseiah (MOS)	Slaughterhouse	24.82754 N	46.79580 E
North (NOS)	Slaughterhouse	24.75572 N	46.66544 E
West (WES)	Slaughterhouse	24.56944 N	46.50776 E
Mansoureiah (MAS)	Slaughterhouse	24.58842 N	46.73466 E
Azizeiah (AZM)	Wholesale market	24.59261 N	46.74299 E
Badea’ah (BAM)	Wholesale market	24.58099 N	46.61288 E
Rabwah (RAM)	Wholesale market	24.69418 N	46.77861 E

**Table 2:** Median lethal doses (LD_50_) and resistance ratio (RR) of *Musca domestica* treated with diazinon.

Population	*N*	LD_50_ (ng/♀)	95% Confidence Limits		Slope ± SE	χ^2^	*p*	RR
			Lower	Upper				
LAB	360	3.27 ^a^	2.83	3.75	2.45 ± 0.24	5.02	0.17	-
RAM	360	204.28 ^b^	163.02	249.74	1.66 ± 0.17	8.79	0.03	62.47
NOS	360	348.22 ^c^	275.56	419.74	2.08 ± 0.24	7.63	0.05	106.48
BAM	360	392.02 ^c^	323.02	496.93	1.95 ± 0.25	7.20	0.06	119.88
AZM	360	405.86 ^c^	333.63	489.53	1.89 ± 0.25	5.48	0.14	124.11
WES	360	452.48 ^c^	381.06	529.63	2.06 ± 0.23	5.90	0.12	138.37
MAS	360	892.26 ^d^	835.11	949.11	5.53 ± 0.53	3.57	0.31	272.86
SAS	360	960.38 ^de^	896.48	1027.58	4.97 ± 0.51	3.22	0.31	293.69
MOS	360	1013.01 ^e^	958.29	1070.32	6.50 ± 0.61	2.93	0.40	309.78

Values that do not share a letter are significantly different where their 95% confidence limits did not overlap.

**Table 3:** Median lethal doses (LD_50_) and resistance ratio (RR) of *Musca domestica* treated with fenitrothion.

Population	*N*	LD_50_ (ng/♀)	95% Confidence Limits		Slope ± SE	χ^2^	*p*	RR
			Lower	Upper				
LAB	360	4.99 ^a^	4.02	6.09	1.71 ± 0.16	0.59	0.90	-
RAM	360	264.88 ^b^	220.71	314.25	2.01 ± 0.18	6.68	0.08	53.08
AZM	360	391.30 ^c^	322.56	481.40	1.68 ± 0.18	8.67	0.03	78.41
BAM	360	481.24 ^c^	416.43	549.59	2.52 ± 0.27	9.48	0.02	96.44
MOS	360	971.15 ^d^	900.00	1047.99	4.37 ± 0.48	1.00	0.80	194.61
MAS	360	1071.92 ^de^	996.52	1161.06	4.49 ± 0.51	5.98	0.11	214.81
NOS	360	1101.06 ^de^	1037.78	1173.12	5.85 ± 0.60	4.44	0.22	220.65
SAS	360	1143.56 ^ef^	1074.29	1226.54	5.51 ± 0.59	0.72	0.87	229.17
WES	360	1303.60 ^f^	1212.77	1431.61	5.18 ± 0.63	0.91	0.82	261.24

Values that do not share a letter are significantly different where their 95% confidence limits did not overlap.

**Table 4:** Activity of AChE in laboratory strain and field populations of houseflies.

Population	Specific Activity (µmol/min/mg Protein)	SD	*p*-Value
LAB	0.161	0.001	
RAM	0.173	0.0008	0.0004
AZM	0.194	0.0004	<0.00001
BAM	0.188	0.0007	0.00001
MOS	0.180	0.0001	0.00004
MAS	0.193	0.0006	<0.00001
NOS	0.191	0.002	0.00002
SAS	0.191	0.0009	0.00001
WES	0.192	0.005	0.0003

**Table 5:** Diazinon median inhibitory concentration (IC_50_) and AChE inhibition resistance ratio (IR).

Population	*N*	IC_50_ (µg/mL)	95% Confidence Limits		Slope ± SE	χ^2^	*p*	IR
			Lower	Upper				
LAB	90	0.77 ^a^	0.60	0.97	1.27 ± 0.19	0.92	0.82	-
WES	90	48.34 ^b^	31.38	63.42	1.53 ± 0.23	1.31	0.73	62.77
RAM	90	63.91 ^bc^	40.43	85.03	1.19 ± 0.20	3.42	0.33	83.00
AZM	90	64.91 ^bc^	48.02	80.44	1.66 ± 0.21	0.43	0.93	84.29
BAM	90	74.05 ^bcd^	53.85	92.91	1.44 ± 0.20	1.76	0.62	96.16
NOS	90	79.41 ^bcd^	60.23	97.69	1.55 ± 0.20	0.15	0.98	103.12
SAS	90	102.26 ^cd^	77.29	128.13	1.30 ± 0.19	4.64	0.20	132.80
MAS	90	108.09 ^d^	91.18	125.97	2.02 ± 0.21	4.04	0.26	140.37
MOS	90	172.59 ^e^	135.17	229.43	1.19 ± 0.19	0.03	1.00	224.14

Values that do not share a letter are significantly different where their 95% confidence limits did not overlap.

**Table 6:** Fenitrothion median inhibitory concentration (IC_50_) and AChE inhibition resistance ratio (IR).

Population	*N*	IC_50_ (µg/mL)	95% Confidence Limits		Slope ± SE	χ^2^	*p*	IR
			Lower	Upper				
LAB	90	0.99 ^a^	0.78	1.20	1.57 ± 0.20	2.01	0.57	-
RAM	90	71.66 ^b^	56.49	85.96	1.90 ± 0.22	3.60	0.31	72.38
AZM	90	79.04 ^b^	58.26	98.73	1.43 ± 0.20	0.59	0.90	79.83
BAM	90	84.53 ^b^	60.85	107.25	1.29 ± 0.20	1.81	0.62	85.38
SAS	90	100.49 ^b^	78.87	122.72	1.50 ± 0.20	4.11	0.25	101.50
MOS	90	104.97 ^bc^	57.58	156.29	0.72 ± 0.18	0.03	1.00	106.03
NOS	90	173.28 ^cd^	127.13	253.73	0.94 ± 0.19	0.89	0.83	175.03
WES	90	214.28 ^d^	172.88	281.99	1.40 ± 0.20	1.24	0.74	216.44
MAS	90	307.76 ^d^	246.25	422.88	1.58 ± 0.21	0.19	0.98	310.86

Values that do not share a letter are significantly different where their 95% confidence limits did not overlap.

## Data Availability

The data presented in this study are available from the corresponding author on a reasonable request.
